# Lack of Detection of Xenotropic Murine Leukemia Virus-Related Virus in HIV-1 Lymphoma Patients

**DOI:** 10.1155/2011/797820

**Published:** 2011-09-08

**Authors:** Krista A. Delviks-Frankenberry, Chawaree Chaipan, Rachel Bagni, Kathleen Wyvill, Robert Yarchoan, Vinay K. Pathak

**Affiliations:** ^1^Viral Mutation Section, HIV Drug Resistance Program, National Cancer Institute at Frederick, National Institutes of Health, Frederick, MD 21702, USA; ^2^Protein Expression Laboratory, SAIC-Frederick Inc., NCI-Frederick, Frederick, MD 21702, USA; ^3^HIV and AIDS Malignancy Branch, National Cancer Institute, National Institutes of Health, Bethesda, MD 20892, USA

## Abstract

Xenotropic murine leukemia virus-related virus (XMRV) is a gammaretrovirus reported to be associated with human prostate cancer and chronic fatigue syndrome. Since retroviruses cause various cancers, and XMRV replication might be facilitated by HIV-1 co-infection, we asked whether certain patients with HIV-associated lymphomas are infected with XMRV. Analysis of PMBCs and plasma from 26 patients failed to detect XMRV by PCR, ELISA, or Western blot, suggesting a lack of association between XMRV and AIDS-associated lymphomas.

## 1. Introduction

A gammaretrovirus, xenotropic murine leukemia virus-related virus (XMRV), was recently discovered and reported to be associated with human prostate cancer (PC) [1]. In the initial report in PC patients, there was a strong correlation between detection of XMRV and a genetic defect in the innate immunity gene *RNASEL * [1]. However, subsequent studies in PC patients showed either no such association [2] or little or no evidence of XMRV infection (reviewed in [3]). Interest in XMRV was increased by the finding that a high percentage of patients with chronic fatigue syndrome (CFS) as well as some asymptomatic patients were infected with XMRV [4]. However, other studies have failed to find such an association (reviewed in [3]). XMRV has been shown to infect peripheral blood mononuclear cells (PBMCs) and CD4+ T cells *in vitro*, indicating that XMRV can infect the same target cells as HIV-1 ([4] and data not shown). However, our recent studies showed that productive replication of XMRV in PBMC and spread in culture are severely restricted by APOBEC3 proteins and perhaps other host defense mechanisms [5]. It remained possible, however, that target cells infected with HIV-1 might provide a favorable environment for XMRV to replicate by depleting cellular host restriction factors such as APOBEC3G, known to inhibit XMRV infection [6, 7]. Several factors led us to explore the possibility that certain patients with HIV-associated lymphoma might be infected with XMRV. Gammaretroviruses can cause lymphomas in other species (reviewed in [8]), and it has been postulated that a number of cases of HIV-associated lymphomas might be caused by an as-yet-unidentified virus [9]. It is also important to note that patients with HIV infection have a higher prevalence of infection with other viruses, and their immunocompromised state might permit more efficient replication of other viruses, including XMRV [9]. Because of the high degree of concern regarding potential XMRV infection and spread in the human population, we sought to investigate whether XMRV might be present in a subset of patients with HIV-associated lymphomas.

## 2. Analysis and Results

**Figure 1:**
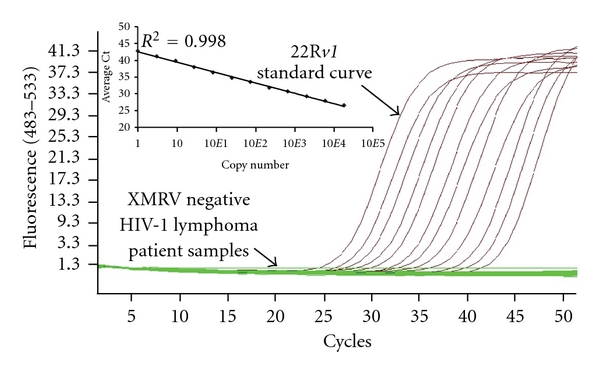
Real-time PCR analysis of HIV-1 lymphoma positive patients for XMRV. XMRV *gag* qPCR failed to detect XMRV from DNA isolated from PBMCs for the 26 HIV-1 lymphoma patients (green lines). Inset shows single-copy sensitivity of the assay.

Total nucleic acids from PBMCs were isolated (Qiagen DNA Blood Mini Kit) from 26 HIV-1 infected patients previously diagnosed with AIDS-associated lymphomas (Table 1). The study was approved by the National Cancer Institutional Review Board, and all patients and donors gave written informed consent. Using a real-time quantitative PCR (qPCR) assay that employed primers specific for a unique 24-nucleotide gap in XMRV *gag* (primer and probe sequences as reported in, [10]), 500 ng of patient DNA was tested in each reaction using the Roche LightCycler 480 Probes Master mix. Cycling conditions using the LightCycler 480 Roche instrument (Roche Diagnostics) were 95°C for 30 sec followed by 50 cycles at 95°C for 15 sec and 60°C for 60 sec. Genomic DNA from the XMRV-expressing cell line 22R*v1* (ATCC) was used to generate a standard curve for XMRV (~20 proviral copies/cell). The standard curve was spiked with 500 ng 293T DNA to ensure similar amplification efficiencies as the patient samples. The qPCR had detection sensitivity of <5 XMRV copies/75,757 cells. The quality and quantity of input DNA were confirmed by detection of human GAPDH by qPCR. Using this assay, all 26 HIV-1 lymphoma patients tested were negative for XMRV *gag* sequences (Figure 1).

**Figure 2:**
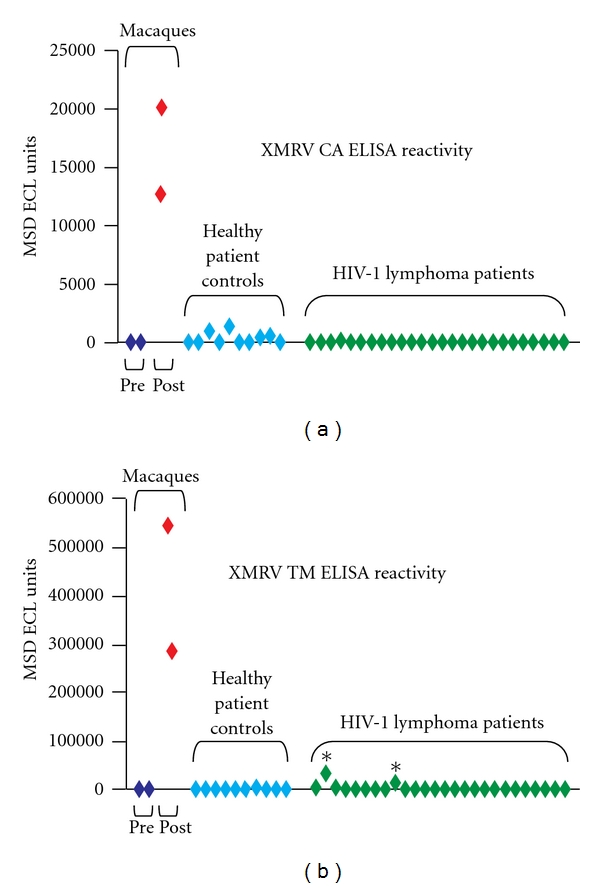
Patient reactivity to XMRV proteins CA and TM. Plasma from the 26 HIV-1 lymphoma positive patients (green diamonds) were assayed by ELISA versus plasma from ten healthy donor controls (light blue diamonds) and two Macaques infected with XMRV pre- (dark blue diamonds) and post- (red diamonds) infection. *indicates two patients with minimal TM reactivity.

Patient plasma was also screened by ELISA (Bagni, Protein Expression Laboratory, SAIC-Frederick Inc., NCI-Frederick, unpublished results) for antibodies against XMRV capsid (CA) and envelope (transmembrane, TM) proteins (Figure 2). Due to the absence of definitive XMRV positive patient control samples, sera from macaques before and after experimental XMRV infection served as controls for baseline and positive reactivity (Lifson and Del Prete, AIDS and Cancer Virus Program, SAIC-Frederick, Inc., unpublished results). Briefly, plasma samples were collected before and after-inoculation (119 days) from two pigtail macaques inoculated with 4.8 × 10^9^ XMRV RNA equivalents derived from 22R*v1* cell supernatants (Lot SP1592, Biological Products Core, AIDS and Cancer Virus Program, SAIC Frederick, Inc., NCI-Frederick). Samples were considered reactive if they were at least 50% reactive relative to the macaque samples following XMRV infection (positive control sera). None of the 26 HIV-1 lymphoma patient, nor the 10 healthy donors, tested positive for the CA (Figure 2(a)) or TM protein (Figure 2(b)), although 2/26 patients had slight reactivity to TM (asterisk, Figure 2(b)). To evaluate these 2 patients, Western blot analysis was performed to assess whether the TM-reactive ELISA test reflected an immune response to XMRV. Endpoint dilution analysis indicated that a 1 : 2000 dilution of the positive macaque sera produced reproducible positive bands on the western blot with a high signal to noise ratio (Lifson and Del Prete, AIDS and Cancer Virus Program, SAIC-Frederick, Inc., unpublished results). Sera from patients (diluted 1 : 50 or 1 : 200) and positive sera from macaques (diluted 1 : 2000) were tested against XMRV viral lysates obtained from the 22R*v1* cell line. The sera from both TM-reactive patients (10- to 40-fold more concentrated than the macaque) failed to detect capsid, TM, or other XMRV proteins (data not shown), indicating that the TM-positive ELISA test was most likely due to the presence of crossreactive nonspecific antibodies.

## 3. Discussion

Our studies show a lack of association between XMRV and AIDS-associated lymphomas and complement other studies that have failed to detect XMRV in the PBMCs, plasma, or seminal plasma from HIV-1 infected patients [11–16]. A potential link between XMRV and cancer is hypothesized in that gammaretroviruses can cause sarcomas and leukemias in rodents, felines, and primates (reviewed in [8]). However, it is unclear whether XMRV infection is associated with prostate or other human cancers. A survey of 800 and 539 PC samples from the United States and Germany, respectively, showed no association with XMRV [17, 18]. A survey of 134 prostate cancer patient plasma samples by us and our collaborators also failed to detect any evidence of XMRV (Kearney et al., under review for this issue of Advances in Virology). Several recent studies have reported that contamination of human samples with mouse DNA may have contributed to XMRV's reported association with human disease [19–22]. Garson et al. [23] also reported that two integration site sequences, previously shown to demonstrate XMRV integration into patient DNA, likely were the result of contamination of identical integration sites from infected DU145 cells reported by the same lab. Furthermore, our recent studies provide compelling evidence that XMRV was generated through recombination of two endogenous murine leukemia viruses during the passage of a PC xenograft in nude mice, and therefore all detection of XMRV from human samples are likely to be due to contamination that originated from this recombination event [24]. It has been hypothesized that this lab-derived virus may have escaped from the lab and is now circulating in the human population. However, our failure to detect XMRV in HIV-1-associated lymphoma patients, along with >10 other studies of different patient cohorts performed by independent investigators, argues against this possibility. 

In Summary, we were unable to detect XMRV DNA or XMRV-specific antibodies in the PBMCs or plasma from HIV-1-associated lymphoma patients, further supporting the absence of a link between XMRV and human cancer.

## Figures and Tables

**Table 1:** HIV-1 Lymphoma Patient Cohort Characteristics.

No. of patients	26
Sex, male/female	24/2
Age, median years (range)	38 (21–58)
CD4 count, cells/mm^3^, median (range)	76 (0–713)
HIV viremic^a^, No. postitive/No. tested	12/21
On anti-HIV drugs	24/26
On AZT	14/26
Lymphomas studied (No. of patients):	PCNSL^b^ (11)
	Diffuse large B cell lymphoma (8)
	Burkitts (1)
	Plasmablastic (1)
	Primary effusion lymphoma (1)
	Primary intraocular (1)
	Hodgkin lymphoma (2)
	Head mass- presumptive PCNSL (1)

^a^Detectable p24 Ag or HIV RNA.

^b^Primary Central Nervous System Lymphoma.
